# Human Gastric Mucins Differently Regulate *Helicobacter pylori* Proliferation, Gene Expression and Interactions with Host Cells

**DOI:** 10.1371/journal.pone.0036378

**Published:** 2012-05-01

**Authors:** Emma C. Skoog, Åsa Sjöling, Nazanin Navabi, Jan Holgersson, Samuel B. Lundin, Sara K. Lindén

**Affiliations:** 1 Mucosal Immunobiology and Vaccine Center, Sahlgrenska Academy, University of Gothenburg, Gothenburg, Sweden; 2 Department of Biomedical Chemistry and Cell Biology, Sahlgrenska Academy, University of Gothenburg, Gothenburg, Sweden; 3 Department of Microbiology and Immunology, Sahlgrenska Academy, University of Gothenburg, Gothenburg, Sweden; 4 Department of Clinical Chemistry and Transfusion Medicine, Sahlgrenska Academy, University of Gothenburg, Gothenburg, Sweden; Veterans Affairs Medical Center (111D), United States of America

## Abstract

*Helicobacter pylori* colonizes the mucus niche of the gastric mucosa and is a risk factor for gastritis, ulcers and cancer. The main components of the mucus layer are heavily glycosylated mucins, to which *H. pylori* can adhere. Mucin glycosylation differs between individuals and changes during disease. Here we have examined the *H. pylori* response to purified mucins from a range of tumor and normal human gastric tissue samples. Our results demonstrate that mucins from different individuals differ in how they modulate both proliferation and gene expression of *H. pylori*. The mucin effect on proliferation varied significantly between samples, and ranged from stimulatory to inhibitory, depending on the type of mucins and the ability of the mucins to bind to *H. pylori*. Tumor-derived mucins and mucins from the surface mucosa had potential to stimulate proliferation, while gland-derived mucins tended to inhibit proliferation and mucins from healthy uninfected individuals showed little effect. Artificial glycoconjugates containing *H. pylori* ligands also modulated *H. pylori* proliferation, albeit to a lesser degree than human mucins. Expression of genes important for the pathogenicity of *H. pylori* (*babA*, *sabA*, *cagA*, *flaA* and *ureA*) appeared co-regulated in response to mucins. The addition of mucins to co-cultures of *H. pylori* and gastric epithelial cells protected the viability of the cells and modulated the cytokine production in a manner that differed between individuals, was partially dependent of adhesion of *H. pylori* to the gastric cells, but also revealed that other mucin factors in addition to adhesion are important for *H. pylori*-induced host signaling. The combined data reveal host-specific effects on proliferation, gene expression and virulence of *H. pylori* due to the gastric mucin environment, demonstrating a dynamic interplay between the bacterium and its host.

## Introduction

Half of the world's population is infected with the bacterium *Helicobacter pylori*. 1–3% of infected individuals develop gastric adenocarcinoma or MALT lymphoma and another 10–15% develop gastritis or gastric and duodenal ulcers, whereas the majority show no symptoms [1]. Adherence of *H. pylori* to the gastric mucosa is highly relevant for the development of gastric disease [2]–[4]. Only part of the colonizing *H. pylori* attaches directly to epithelial cells [5]. Instead, most of them live in the mucus layer of the superficial gastric mucosa where they can bind to the highly glycosylated mucins [6], [7]. The mucus layer in the stomach consists mainly of the secreted mucins MUC5AC, produced from the superficial mucosa, and MUC6, produced from the gland mucosa [8]. The best characterized *H. pylori* adhesins are the blood group antigen binding adhesin (BabA) that binds to Lewis b (Le^b^) and H-type 1 structures [9], and the sialic acid binding adhesin (SabA) that binds to sialyl-Le^x^ and sialyl-Le^a^ [10]. The mucus layer protects the gastric mucosa by acting as a physical barrier preventing *H. pylori* from binding to the epithelia [7], [11]. Furthermore, terminal 1,4-linked N-acetylglucosamine (α1,4-GlcNAc), which is a carbohydrate structure on mucins present in the gastric glands, has been demonstrated to have antimicrobial activity [12], which may contribute to protection against *H. pylori* colonization of the gastric glands.

In *H. pylori* infection, precancerous lesions and cancer, the expression, glycosylation and spatial distribution of mucins change both in humans and in the rhesus monkey infection model [11], [13]–[17]. As a consequence, the *H. pylori* adhesion targets and the glycan environment that *H. pylori* is exposed to can dramatically change [7], [11], [17]. For example, in the rhesus monkey there are time-dependent changes in the Le^b^ expression and induced expression of sialylated Lewis antigens upon infection with *H. pylori* [11], and in humans, high level of Le^a^ and sialylation is associated with gastric tumors [16], [18].

The effect of the mucins and the change in mucin environment on *H. pylori* during infection and associated disease is poorly understood. Crude porcine gastric mucins have been shown to stimulate proliferation of *H. pylori* [19], [20], and antral surface mucins from normal tissue of one gastric cancer patient stimulate *H. pylori* proliferation [12]. In addition, the intestinal pathogen *Campylobacter jejuni* responds to human MUC2 with decreased proliferation and altered gene expression [21].

In this study, we investigated how *H. pylori* can be affected by mucins from different individuals and disease states. We examined the interactions between *H. pylori* and mucins regarding binding, proliferation, gene expression and virulence of *H. pylori* when exposed to purified mucins from a range of gastric mucosal samples from healthy and gastric cancer affected individuals. We found that mucins from different individuals could diversely modulate *H. pylori* behavior in all these aspects, partly dependent on mucin origin and *H. pylori* binding ability.

## Results

### Mucins isolated from different regions, individuals and disease stages differ in glycosylation

We isolated mucins from normal gastric mucosa from healthy individuals and gastric tumor tissue as well as normal gastric mucosa from tumor-affected stomachs (Table 1). The mucin and carbohydrate contents of the samples are summarized in Table 2. The normal gastric mucosa isolated from tumor-affected stomachs was divided into surface versus gland material and thereby MUC5AC was separated from MUC6. Mucins were further divided into those soluble and insoluble in guanidinium hydrochloride (GuHCl): Insoluble mucins were mainly MUC2, whereas most of the MUC5AC and MUC6 were present in soluble fractions. MUC2 was only found in tumor samples. Unlike samples from macroscopically normal tissue, tumor mucin samples contained sialyl-Le^x^ and sialyl-Le^a^ in concordance with previously published results that sialylation of gastric tissue mucins is associated with cancer [16]. The lack of MUC2, MUC5B, superficial MUC6 and sialylation of non-tumor samples confirms the pathologist's report stating that these specimens were normal. It has previously been reported that terminal α1,4-GlcNAc is co-localized with MUC6 in the glands [22], [23]. In agreement with this, the mucins isolated from the gland mucosa contained more α1,4-GlcNAc than mucins isolated from the superficial mucosa. However, the tumor sample containing MUC6 did not contain α1,4-GlcNAc (Table 2).

**Table 1:** Origin, short names and density of the mucin samples used in this study.

Origin	Patient number and sample type	Short name	Density (g/l)
**Gastric tumor mucosa**	Patient 1 Tumor soluble	P1 TS	1.39–1.43
	Patient 1 Tumor insoluble (high density)	P1 TI (hd)	1.40–1.44
	Patient 1 Tumor insoluble (low density)	P1 TI (ld)	1.36–1.38
	Patient 2 Tumor soluble	P2 TS	1.35–1.43
	Patient 3 Tumor soluble	P3 TS	1.35–1.45
	Patient 4 Tumor soluble (high density)	P4 TS (hd)	1.41–1.45
	Patient 4 Tumor soluble (low density)	P4 TS (ld)	1.33–1.39
	Patient 4 Tumor insoluble	P4 TI	1.32–1.48
**Normal mucosa of tumor-affected stomachs**	Patient 5 Tumor-adjacent antrum surface	P5 TA-AS	1.36–1.42
	Patient 5 Tumor-adjacent antrum glands	P5 TA-AG	1.34–1.41
	Patient 6 Tumor-adjacent fundus surface	P6 TA-FS	1.36–1.43
	Patient 6 Tumor-adjacent fundus glands	P6 TA-FG	1.36–1.42
	Patient 6 Tumor-adjacent antrum surface	P6 TA-AS	1.36–1.42
	Patient 6 Tumor-adjacent antrum glands	P6 TA-AG	1.38–1.42
	Patient 7 Tumor-adjacent antrum surface	P7 TA-AS	1.34–1.38
	Patient 7 Tumor-adjacent antrum glands	P7 TA-AG	1.36–1.42
**Healthy gastric mucosa**	Patient 8 Healthy	P8 Healthy	1.37–1.42
	Patient 9 Healthy	P9 Healthy	1.37–1.42
	Patient 10 Healthy	P10 Healthy	1.35–1.41

**Table 2:** Content of mucins and glycan structures in isolated mucin samples.

Origin	Sample	MUC5AC	MUC6	MUC2	MUC5B	Le^b^	Sialyl-Le^x^	Sialyl-Le^a^	α1,4-GlcNAc
**Gastric tumor mucosa**	P1 TS	+	++	−	++	++	++	+	−
	P1 TI (hd)	−	−	+	−	++	++	+	−
	P1 TI (ld)	−	−	+	−	+	++	−	−
	P2 TS	+	−	+	−	+	+	++	−
	P3 TS	+	−	−	−	+	+	−	−
	P4 TS (hd)	−	−	+	−	+	+	−	−
	P4 TS (ld)	−	−	+	−	++	+	−	−
	P4 TI	−	−	++	−	+	−	−	−
**Normal mucosa of tumor-affected stomachs**	P5 TA-AS	+	−	−	−	++	−	−	−
	P5 TA-AG	++	+	−	−	++	−	−	+
	P6 TA-FS	++	−	−	−	++	−	−	+
	P6 TA-FG	++	+	−	−	++	−	−	++
	P6 TA-AS	+	−	−	−	++	−	−	+
	P6 TA-AG	+	+	−	−	++	−	−	++
	P7 TA-AS	++	−	−	−	++	−	−	−
	P7 TA-AG	+	+	−	−	++	−	−	+
**Healthy gastric mucosa**	P8 Healthy	+	−	−	−	−	−	−	−
	P9 Healthy	+	−	−	−	++	−	−	−
	P10 Healthy	+	−	−	−	++	−	−	+

The relative amount of mucins and glycan structures relevant for interactions with *H. pylori* were determined by ELISA. The relative presence or absence of each structure is indicated with ++ (above 75% of the highest assay value), + and − (OD_450_ less than 0.15 for antibodies giving a high signal and 0.1 for antibodies giving a low signal). Abbreviations of sample identity are explained in Table 1.

### Proliferation of *H. pylori* varies in the presence of mucins of different origin

**Figure 1:**
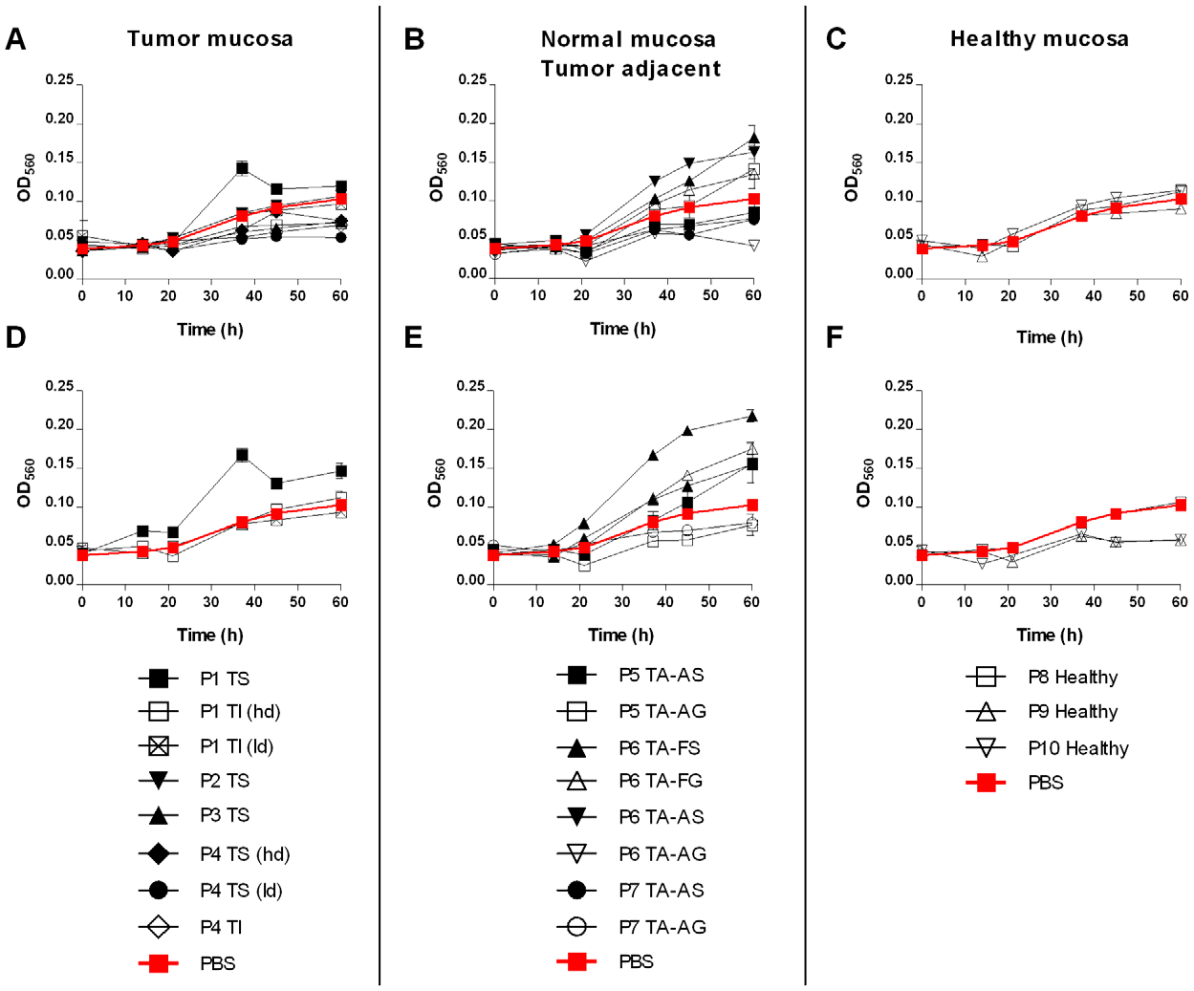
Proliferation of *H. pylori* J99 in the presence of purified mucins. Proliferation of *H. pylori* J99 wt during 60 h in the presence of mucins derived from: A) gastric tumor mucosa at 10 µg/mL (closed symbols = soluble mucins, open symbols = insoluble mucins), B) normal mucosa of tumor-affected stomachs at 10 µg/mL (closed symbols = surface mucins, open symbols = gland mucins), C) healthy mucosa at 10 µg/mL, D) gastric tumor mucosa at 50 µg/mL, E) normal mucosa of tumor-affected stomachs at 50 µg/mL and E) healthy mucosa at 50 µg/mL. Control without mucins (PBS control) is shown in bold red. An OD_560_ of 0.1 corresponds to 10^7^ CFU/mL.

**Figure 2:**
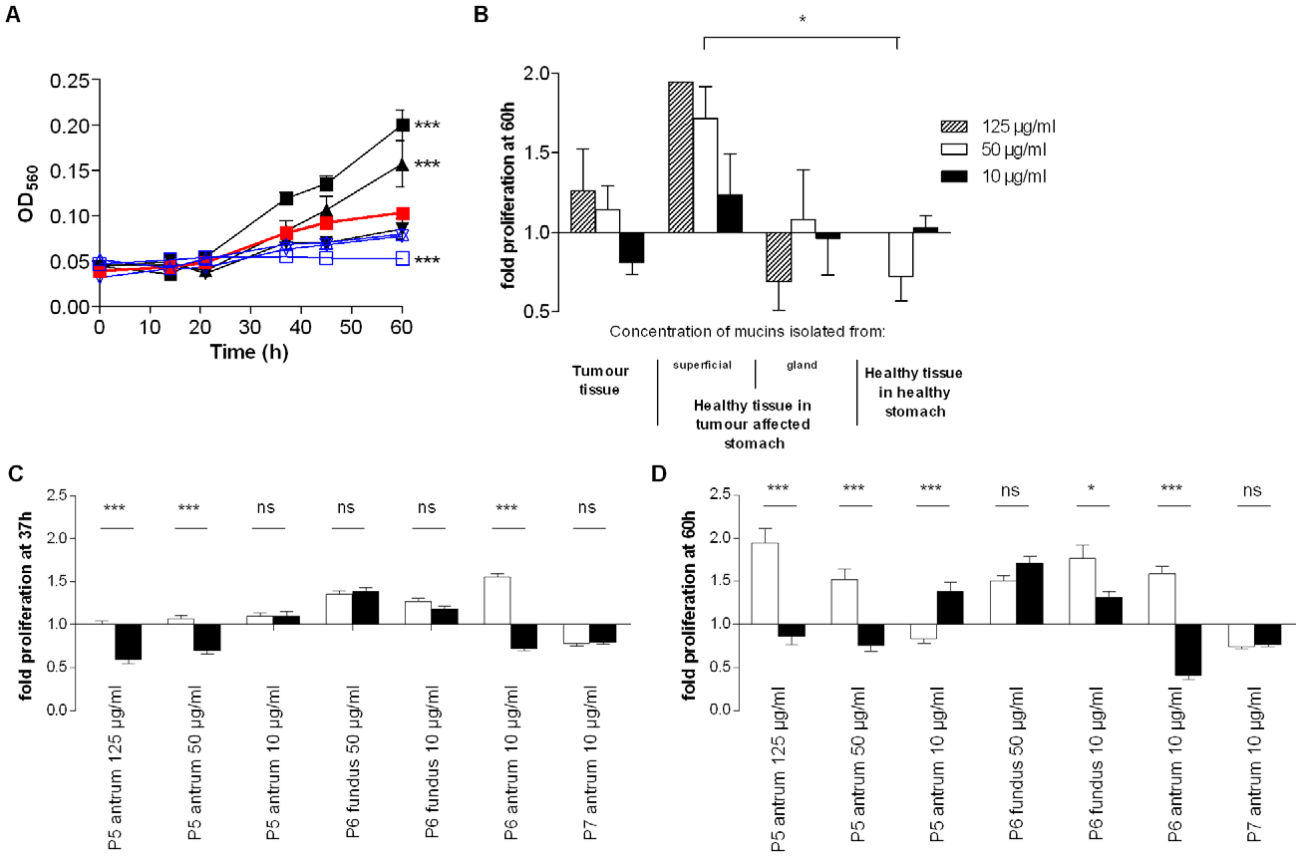
Concentration- and tissue-dependency of purified mucins for *H. pylori* J99 proliferation. A) Concentration-dependency of one stimulatory (P5 TA-AS, black closed symbols) and one inhibitory mucin sample (P7 TA-AG, blue open symbols) on *H. pylori* proliferation comparing mucin concentrations of 125 (▪), 50 (▴) and 10 (▾) µg/mL to proliferation without mucins (bold red ▪). (***p≤0.001, One-way ANOVA, Dunnett t-test, compared to J99 without mucins after 60 h proliferation.) B) *H. pylori* fold proliferation level compared to PBS control after 60 h with mucins grouped by tissue type origin at 125, 50 and 10 µg/mL of mucin. (*p = 0.046, One-way ANOVA, Tukey's post hoc test.) C and D) *H. pylori* fold proliferation level compared to PBS control after 37 h and 60 h with mucins from superficial (white bars) or gland (black bars) mucosal tissue from the same individual. (***p≤0.001, *p<0.05, ns = not significant, Student's t-test.) Although the differences between gland and surface derived mucins was larger at 60 h than at 37 h, some of the growth curves had lost their parabolic growth by this time, which explains why the results for one sample is different at 60 h than at 37 h.

*H. pylori* J99 wt was cultured in liquid media in the presence of mucins samples to monitor effects of mucins on *H. pylori* proliferation. The effects of all mucins were analyzed at a concentration of 10 µg/mL, whereas fewer samples were analyzed at higher concentrations since not all gastric specimens obtained were of sufficient size to yield large amounts of mucins. Mucins isolated from different individuals differed in their effect on *H. pylori* proliferation (p≤0.001, One-way ANOVA) and ranged from 86% inhibition to 121% increase in proliferation after 60 h culture with 10 µg/mL mucin samples and from 76% inhibition to 175% increase with 50 µg/mL of mucins (Figure 1). The effect on both inhibition and stimulation of proliferation increased with higher mucin concentrations (Figure 1, Figure 2A). At 50 µg/mL, *H. pylori* proliferation was higher in the presence of tumor-adjacent surface tissue mucins than with mucins from healthy stomachs (Figure 2B). In general, mucins from healthy stomachs had no or inhibitory effect, whereas mucins from diseased stomachs varied greatly in their ability to affect *H. pylori* proliferation. The mucins with the most stimulatory effect were isolated from tumor-adjacent surface tissues and from one of the tumors (P1 TS). However, the substantial variation within each group indicates that interindividual factors other than disease state are important.

*H. pylori* proliferation was higher in the presence of the superficial mucins than with the gland mucins from the same individual and stomach location in 2 of 4 cases after 37 h and in 3 of 4 cases after 60 h culture (Figure 2C and D). This is in line with a previous study where gland mucins from one patient inhibited proliferation due to the presence of α1,4-GlcNAc and superficial mucins from the same patient stimulated proliferation [12]. However, the gland mucins from fundus did not inhibit proliferation in our study even though they contain high amounts of α1,4-GlcNAc. Mucin and mucin glycan content from healthy stomachs were similar to that of mucins isolated from tumor-adjacent surface tissue mucins. The difference in effect on proliferation (Figure 2B) between these two groups of mucins are thus not dependent on α1,4-GlcNAc.

### Binding ability of *H. pylori* J99 varies between mucin samples and mucins that induce most proliferation show strong adherence to *H. pylori*

**Figure 3:**
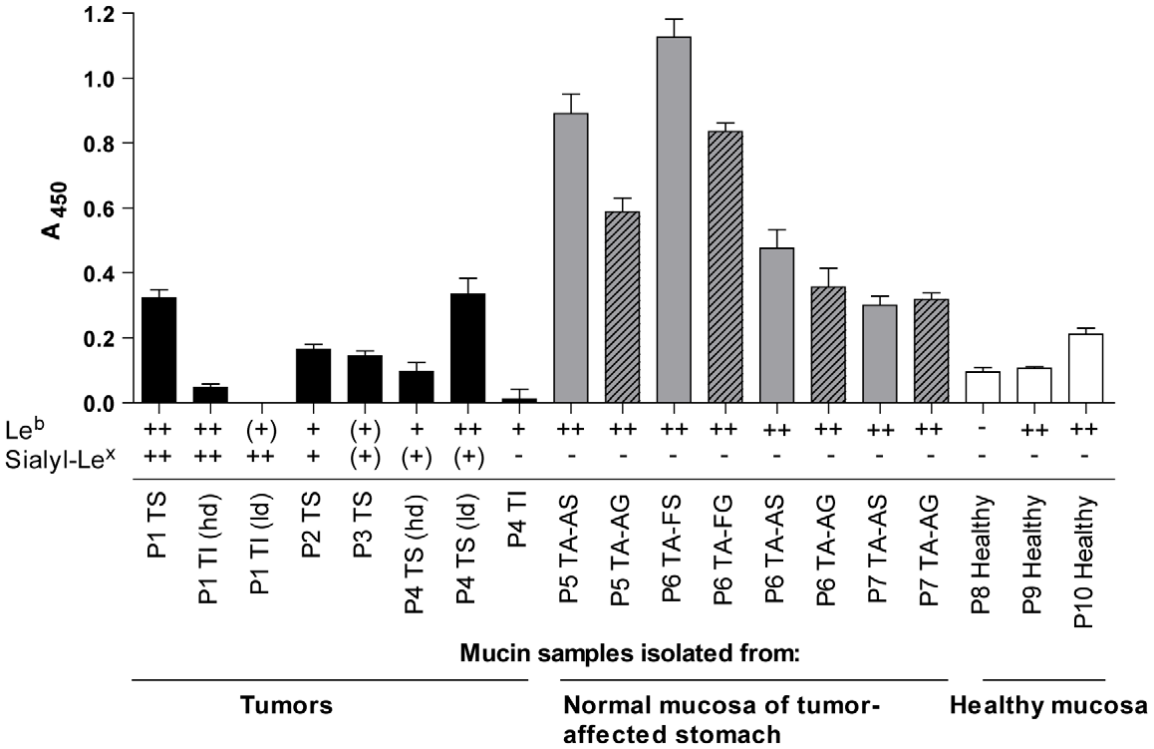
Binding of *H. pylori* J99 to mucin samples. Binding of biotinylated *H. pylori* J99 to mucin samples was measured in a microtiter-based assay. The presence or absence of Le^b^ and sialyl-Le^x^ in each mucin samples, as described in Table 2, is indicated below the x-axis.

Similar to the proliferation response, *H. pylori* binding ability to surface mucin samples was higher than that to gland mucin samples from the same patient for most samples (Figure 3). Binding correlated with the presence of MUC5AC and Le^b^ (*r* = 0.738, p≤0.001 and *r* = 0.516, p = 0.024, Pearson's correlation), but not with sialyl-Le^x^ or sialyl-Le^a^, in concordance with previously published data demonstrating that *H. pylori* binds mainly to MUC5AC-associated Le^b^ at neutral pH [6]. Mucins isolated from healthy stomachs and tumors tended to be less potent ligands for *H. pylori* J99 than mucins from tumor-adjacent tissue, even when MUC5AC and Le^b^ amounts were similar. Thus, the *H. pylori* binding ability of the mucins also varied depending of uncharacterized individual differences, most likely relating to the steric presentation of glycans. There was no binding to the insoluble mucins isolated from the tumor samples (Figure 3), which contain mainly MUC2 and no MUC5AC. The ability of *H. pylori* to bind mucins seems to be associated to proliferation as most mucin samples that bound to *H. pylori* tended to induce proliferation (Pearson's correlation between *H. pylori* binding and proliferation level at 60 h with mucins at 50 µg/mL: *r* = 0.562, p = 0.057).

**Figure 4:**
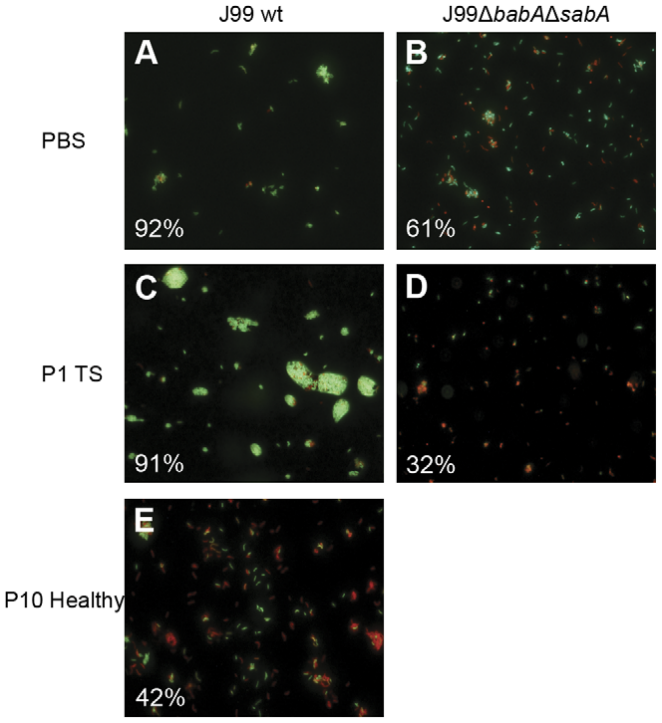
Aggregation and viability of *H. pylori* J99 after culture with mucins. *H. pylori* were stained with LIVE/DEAD® BacLight™ bacterial viability staining after 37 h culture with or without 50 µg/mL of mucin samples. Green represents bacteria with intact membranes and red represents dead bacteria or bacteria with damaged membranes. The percentages of live bacteria are noted in each picture. A) J99 wt with PBS, B) J99Δ*babA*Δ*sabA* with PBS, C) J99 wt with patient 1 soluble tumor mucins, D) J99Δ*babA*Δ*sabA* with patient 1 soluble tumor mucins and E) J99 wt with patient 10 healthy gastric mucins.

In addition to the binding analyzed by the microtiter-based assay, binding to mucins in the environment of the proliferation assay as well as the bacterial viability was visualized with LIVE/DEAD® BacLight™ bacterial viability staining. Alike most other stimulatory mucin samples, *H. pylori* strain J99 wt cultured with the patient 1 soluble tumor mucins showed aggregates of highly viable mucin-binding *H. pylori* compared to the less aggregated *H. pylori* without mucins (Compare Figure 4A and C). This indicates that the patient 1 soluble tumor mucins bind strongly to *H. pylori* in the soluble environment that the proliferation assay is performed in even though binding to these mucins when attached to a microtitre well surface (Figure 3) were intermediate/low. *H. pylori* J99 wt cultured with inhibitory mucins showed less or no aggregating bacteria and also showed a lower ratio of live bacteria with intact membranes after 37 hours of culture (Figure 4E). These results support that binding is involved in the proliferation response to mucins and that some mucin samples act inhibitory on *H. pylori*.

### Le^b^ glycoconjugates induce more *H. pylori* proliferation than sialyl-Le^x^ glycoconjugates

**Figure 5:**
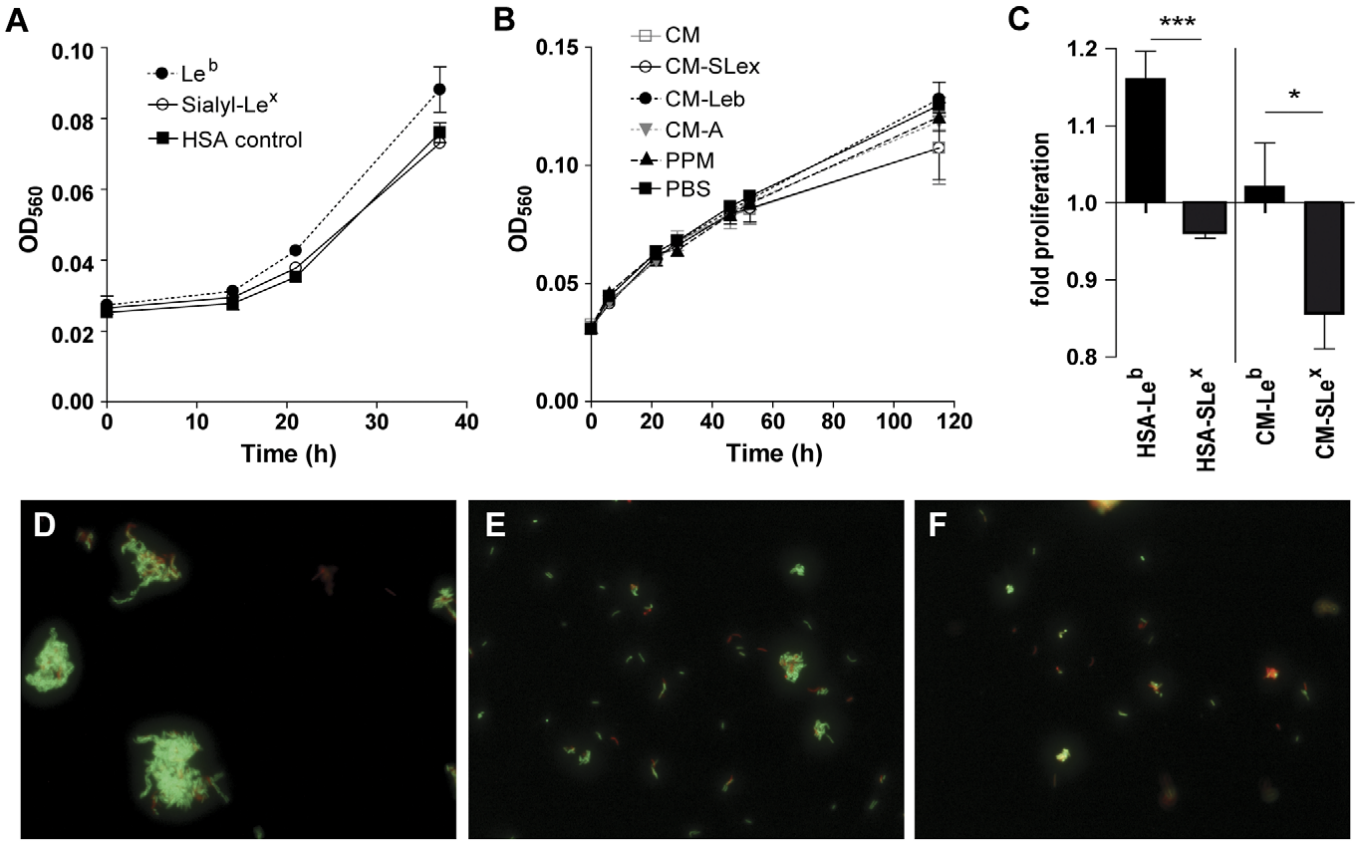
Proliferation of *H. pylori* J99 in the presence of HSA-glycoconjugates and recombinant mucins. A) Proliferation of *H. pylori* J99 wt cultured with 50 µg/mL of Le^b^ or sialyl-Le^x^ glycoconjugate and HSA control. B) Proliferation of *H. pylori* J99 wt cultured with 50 µg/mL of recombinant PSGL-1. CM = unmodified CHO-K1 PSGL-1 containing mono- and disialylated core 1, CM-SLe^x^ = sialyl-Le^x^ substituted PSGL-1, CM-Le^b^ = Le^b^ substituted PSGL-1, CM-A = blood group A active PSGL-1 and PPM = mannose-containing PSGL-1 lacking sialic acid produced in the yeast Pichia pastoris. C) Fold proliferation level of *H. pylori* J99 wt in the presence of HSA-glycoconjugates compared to HSA control after 37 h and recombinant PSGL-1 compared to PBS control after 115 h. ***p≤0.001, *p<0.05, Student's t-test. D-F) Bacteria were stained with LIVE/DEAD® BacLight™ bacterial viability kit after culture with D) HSA-Le^b^ conjugate, E) HSA-sialyl-Le^x^ conjugate and F) HSA control. Green represents bacteria with intact membranes and red represents dead bacteria or bacteria with damaged membranes.

*H. pylori* J99 wt was cultured in the presence of Le^b^ and sialyl-Le^x^ glycoconjugates to isolate the effect of these *H. pylori* ligands on proliferation. The Le^b^ and sialyl-Le^x^ structures were presented as hexamers, conjugated to human serum albumin (HSA) to provide multivalency. *H. pylori* proliferation after culture with the Le^b^ conjugate was increased compared to that of the HSA control and sialyl-Le^x^ conjugate (Figure 5A, p≤0.001, One-Way ANOVA). Aggregation of bacteria occurred after culture with Le^b^ conjugate (Figure 5D), whereas less and smaller aggregates were seen with the sialyl-Le^x^ conjugate or in the control (Figure 5E and F, respectively), supporting previously published data that *H. pylori* J99 wt bound to the Le^b^ conjugate [24].

To more closely resemble the glycan presentation to that of gastric mucins, *H. pylori* J99 wt was cultured with the recombinant mucin-type glycoprotein P-selectin glycoprotein ligand-1 (PSGL-1) with variable glycosylation expressed in CHO-K1 and yeast cells [25]–[27]. A small difference in proliferation could be seen first after 3–4 days (Figure 5B). Similar to the results with HSA-glycoconjugates, *H. pylori* proliferation was higher in the presence of PSGL-1 with Le^b^ than PSGL-1 with sialyl-Le^x^ (Figure 5C). Together these results indicate that the binding to Le^b^, but not to sialyl-Le^x^, leads to an increase in proliferation. Both glycoproteins containing sialic acid; with mono-and disialylated core 1 or with sialyl-Le^x^, gave slightly lower proliferation than that of *H. pylori* cultured without glycoproteins, whereas there were no change in proliferation with glycoproteins without sialic acid (Figure 5B). However, neither HSA-glycoconjugates nor recombinant mucin-type glycoproteins were able to induce as major changes in proliferation as seen after culture with human mucin samples.

### Other *H. pylori* strains also display a binding-dependent proliferation response to mucins

To test whether the effect on mucins on *H. pylori* is relevant to other strains, additional *H. pylori* strains with different mucin binding capabilities were cultured in the presence of mucin samples that showed stimulatory and inhibitory effects on J99 wt proliferation. Strains CCUG17875/Le^b^, HP 201 and HP 1172 which have a similar mucin binding ability as strain J99 wt [28], also enhanced proliferation in response to the patient 1 soluble tumor mucins (Table 3). The strains that in previous studies showed little or no BabA-mediated binding to mucins (strains HP 364, J99Δ*babA*Δ*sabA*, 26695 and CCUG17874 [7], [10], [28]), did not show increased proliferation in response to the patient 1 soluble tumor mucins (Table 3). The J99 adhesin deletion mutant J99Δ*babA*Δ*sabA* had a higher baseline proliferation and lower proportional viability than its parent strain (Figure 4B). Microscopy studies of J99Δ*babA*Δ*sabA* verified the lack of binding to the mucin sample (i.e. lack of bacterial aggregation after mucin treatment) and also showed less viable bacteria after culture with mucins (Figure 4D). Strain CCUG17874 bind to the patient 1 soluble tumor mucins via SabA and not BabA [7], [28]. Therefore the lack of increased proliferation in response to this mucin further supports that binding to Le^b^, but not to sialyl-Le^x^, plays a role for the proliferation response. In summary, the strains that bind mucins via BabA (CCUG17875/Le^b^, HP 201, HP 1172 and J99 wt) showed enhanced proliferation in culture with the stimulatory mucin, whereas strains that do not have the BabA adhesin did not (strains HP 364, J99Δ*babA*Δ*sabA*, 26695 and CCUG17874), irregardless of if they carry the SabA adhesin or not. Thus, other strains than J99 also respond to mucins by a change in proliferation. The mucin binding ability via BabA seems to be an important factor for enhancing the *H. pylori* proliferation in response to mucins, whereas inhibition of proliferation in response to the mucins that have an inhibiting effect appear to be independent of adhesion to mucins.

**Table 3:** Proliferation of different *H. pylori* strains in the presence of two mucin samples.

	P1 tumor soluble	P7 TA antrum gland
J99 wt	206±18***	76±5***
J99ΔbabAΔsabA	105±10	89±6***
26695	99±25	99±14
CCUG17874	100±7	93±5*
CCUG17875/Le^b^	110±6**	85±5***
HP201	436±27***	nd
HP364	87±11	nd
HP1172	156±6	nd

Effect of mucins (50 µg/mL) on proliferation of different *H. pylori* strains expressed as percentage of proliferation without mucins. (***p≤0.001, **p<0.01, *p<0.05, Independent Student's t-test, nd = not determined).

### Gene expression of *H. pylori* cultured with mucins co-varies

**Figure 6:**
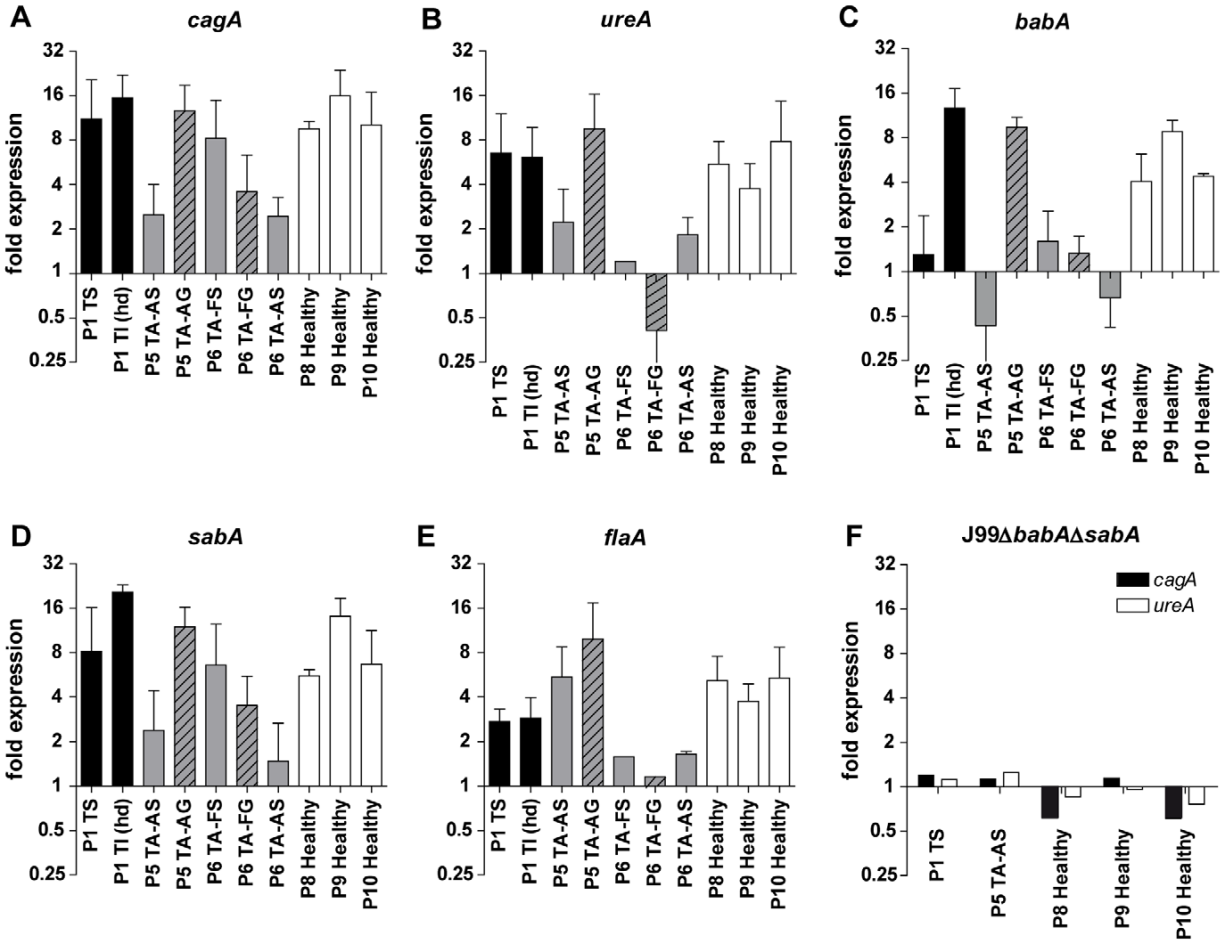
Gene expression of *H. pylori* in response to mucins. A–E) The gene expression of *cagA*, *ureA*, b*abA*, *sabA* and *flaA* of *H. pylori* J99 wt cultured in the presence of 50 µg/mL mucin samples derived from tumor tissue (black bars), normal mucosa of tumor-affected stomachs (grey; filled bars = surface mucins, striped bars = gland mucins) and healthy mucosa (white bars). Expressions are shown as fold expression to *H. pylori* cultured without mucins, normalized to 16S rRNA. A fold expression difference greater than 4 is considered significant. F) J99Δ*babA*Δ*sabA* fold expression of *cagA* and *ureA*.

As mucins differently affect *H. pylori* proliferation we hypothesized that mucins also can modulate the expression of genes involved in mucin binding and other virulence processes. *H. pylori* response to mucins at the transcriptional level was analyzed by real-time PCR and the expression of *babA*, *sabA*, *flaA*, *cagA*, and *ureA* was normalized to the expression of 16S rRNA. Mucin samples were able to modulate the expression of *H. pylori* J99 wt genes at different extents (Figure 6A–E), but not for J99Δ*babA*Δ*sabA* (Figure 6F). The genes *babA*, *sabA* and *cagA* from *H. pylori* J99 showed the same expression level pattern for each mucin sample with intercorrelation between the genes (r = 0.85–0.93, p≤0.001), which was also found between ureA and flaA (r = 0.74, p = 0.01, Table 4) and which indicates that these genes are regulated by common pathways in the response to mucins. None of the samples that increase the *babA* expression were able to stimulate *H. pylori* proliferation. The expression of *babA* and *ureA* correlated negatively to proliferation levels at 60 hours (*r* = 0.684–0.705, p<0.05), and the expression of *cagA* correlated negatively both to proliferation and mucin binding (*r* = 0.789, p<0.01 and *r* = 0.639, p<0.05). These results suggest that weak interactions to mucins increase expression of *H. pylori* adhesin and virulence factors as opposed to when there is high binding to mucins associated with enhanced proliferation or when there is no binding to mucins.

**Table 4:** Correlation between gene expression levels.

		*babA*	*sabA*	*cagA*	*flaA*
***babA***	Pearson Correlation	1			
	p-value				
***sabA***	Pearson Correlation	0.934	1		
	p-value	0.000			
***cagA***	Pearson Correlation	0.851	0.914	1	
	p-value	0.001	0.000		
***flaA***	Pearson Correlation	0.405	0.199	0.302	1
	p-value	0.216	0.557	0.366	
***ureA***	Pearson Correlation	0.601	0.535	0.665	0.736
	p-value	0.050	0.090	0.026	0.010

Correlation of expression of virulence genes in *H. pylori* J99 co-cultured with different mucin preparations.

### Mucin-pretreatment of *H. pylori* inhibits binding to gastric epithelial cells and protects their viability

**Figure 7:**
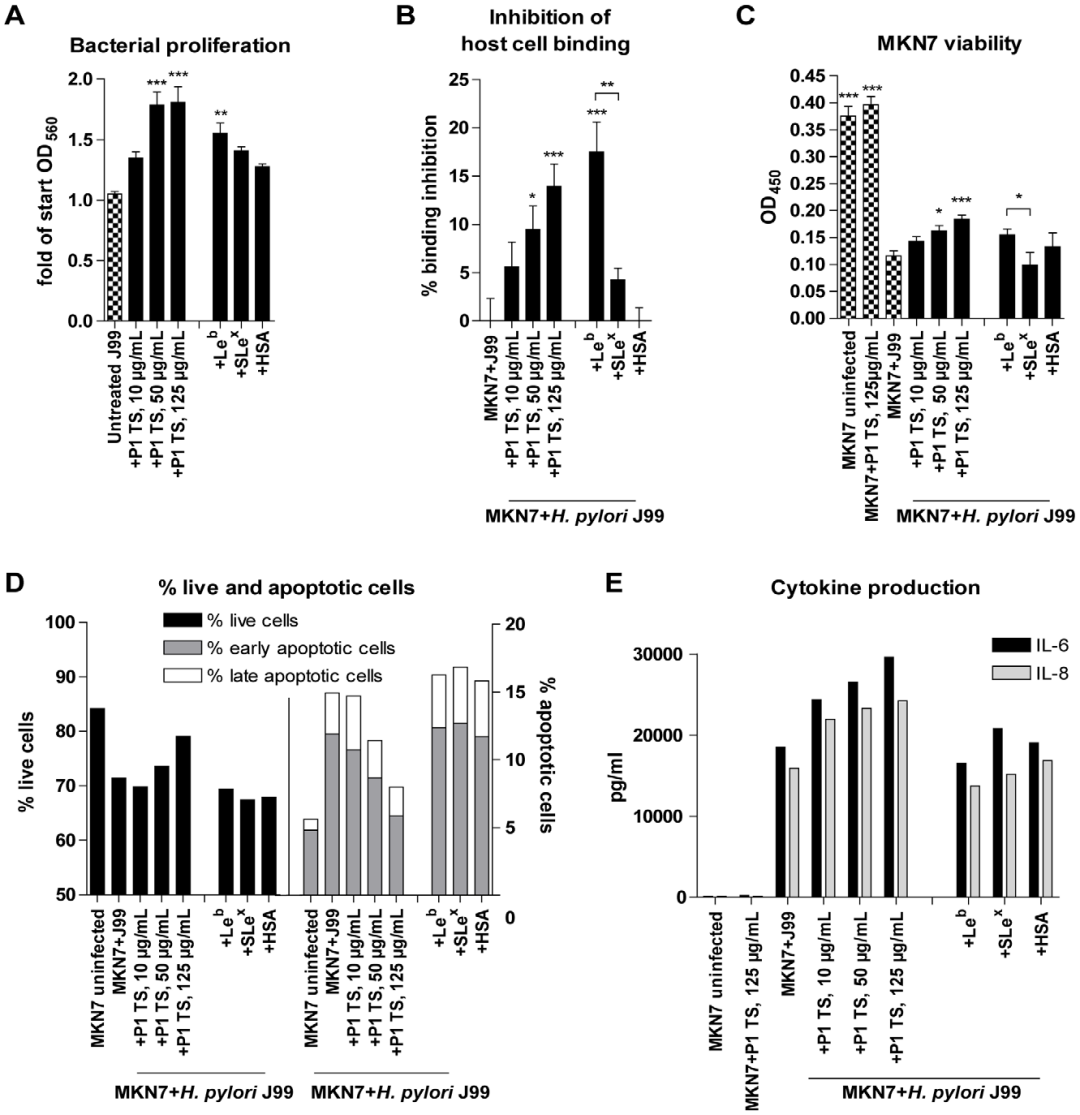
Effects on mucin concentration and host cell adhesion on viability and cytokine production after infection. A) *H. pylori* J99 wt proliferation with patient 1 soluble tumor mucins and 50 µg/mL glycoconjugates after 24 h culture in RPMI 10% FBS, prior to MKN7 infection, expressed as fold change from start of culture. B) Binding of *H. pylori* to MKN7 cells after 24 h infection expressed as percentages less binding than untreated *H. pylori*. C) Viability of MKN7 cells measured by cell metabolic activity (WST-1 assay) after 24 h infection. Data are shown after subtracting the small signal produced by the presence of bacteria in the assay. D) Percentage of live, early and late apoptotic cells after 24 h infection measured by flow cytometry (Annexin V/7AAD). E) Concentration of IL-6 and IL-8 in cell culture media after 24 h infection (One out of two experiments carried out at different concentrations with similar results are shown). (***p≤0.001, **p<0.01, *p<0.05, ANOVA, Dunnett t-test, compared to J99 without mucins or with HSA control. Independent Student's t-test was used to compare between Le^b^ and sialyl-Le^x^ glycoconjugates.)

Our results that both proliferation and gene expression can be modulated by mucins indicate that mucins affect the virulence of *H. pylori*. This was investigated by infecting an *in vitro* infection model of confluent gastric MKN7 cells, mimicking the gastric epithelial surface cell layer, with *H. pylori* pretreated with the mucin samples. The mammalian cell culture conditions (RPMI 1640 with 10% FBS) supported concentration-dependent effects of mucins on *H. pylori* proliferation similar to the effects in BHI with 10% FBS (Figure 7A). Patient 1 soluble tumor mucins inhibited binding of *H. pylori* to MKN7 cells in a concentration-dependent manner (Figure 7B). The metabolic activity (as measured with the WST-1 reagent) of the MKN7 cells decreased after co-culture with *H. pylori* and this decrease was partially restored when *H. pylori* was pretreated with this mucin sample (Figure 7C). In line with these results, the flow cytometry apoptosis assay (Annexin V/7AAD) showed an increased number of live MKN7 cells and a decreased number of apoptotic cells with higher concentration of the patient 1 soluble tumor mucins added to *H. pylori* precultures compared to without mucins (Figure 7D). Inhibition of bacterial binding to the cells by mucins thus protected their viability. Similarly, *H. pylori* pretreated with the Le^b^-HSA-conjugate inhibited *H. pylori* binding to the cells and increased cell viability to a higher extent than the sialyl-Le^x^-HSA conjugate (Figure 7B and C). Although Le^b^-HSA-conjugate strongly inhibited *H. pylori* binding to the cells, it did not protect the cell viability as much as patient 1 soluble tumor mucins. Thus, limiting adhesion is not the only manner in which mucins protect gastric epithelial cell viability during infection.

**Figure 8:**
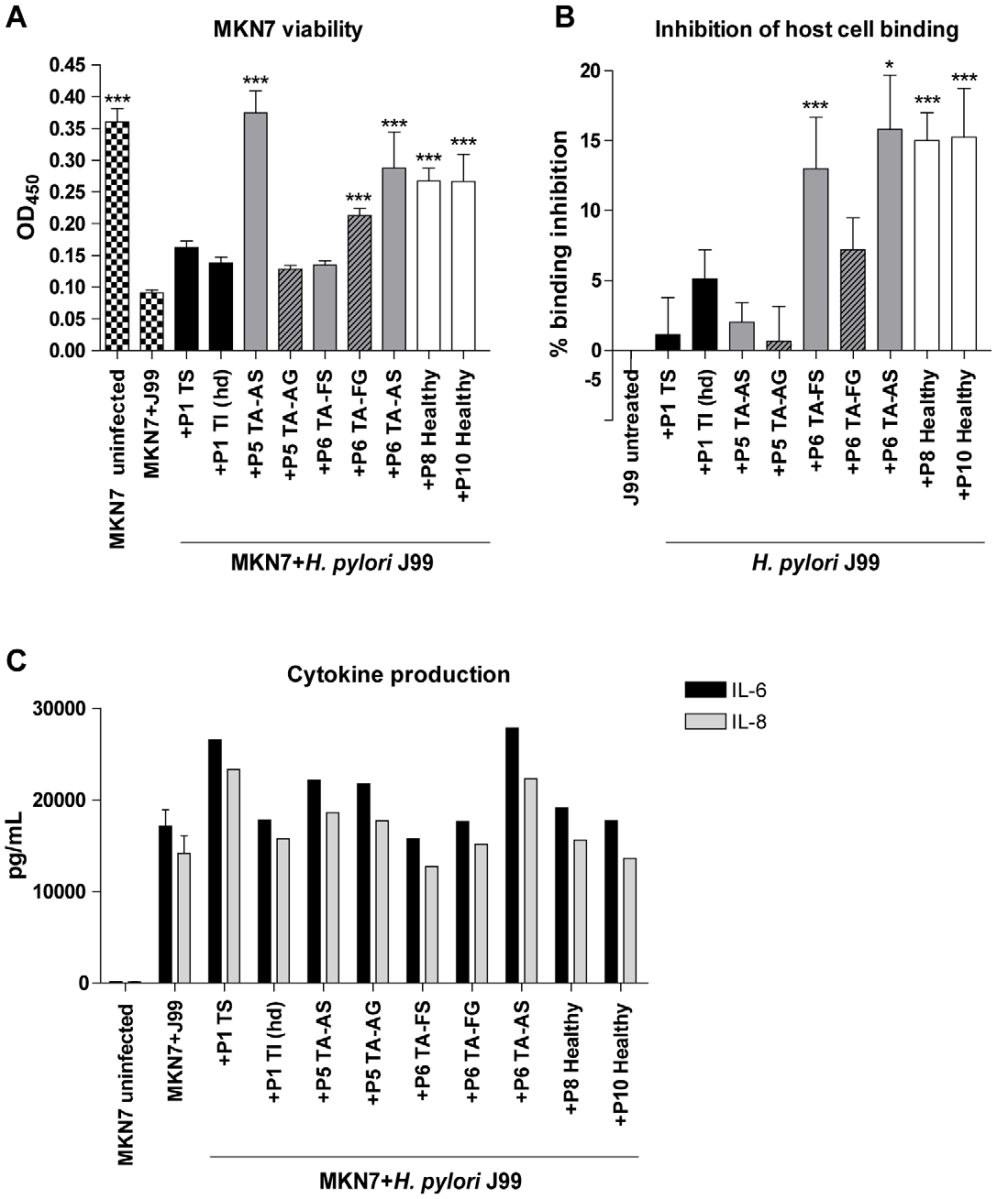
Host cell adhesion, viability and cytokine production after infection with *H. pylori* pretreated with different mucins. A) Viability of MKN7 cells measured by the cell proliferation reagent WST-1 after 24 h infection with *H. pylori* pretreated with mucin samples derived from tumor tissue (black bars), normal mucosa of tumor-affected stomachs (grey; filled bars = surface mucins, striped bars = gland mucins) and healthy mucosa (white bars), compared to controls without mucins (black and white bars). B) Binding of *H. pylori* to MKN7 cells after 24 h infection expressed as percentages less binding than untreated *H. pylori*. C) Concentration of IL-6 and IL-8 in cell culture media after 24 h infection (One out of two experiments carried out at different concentrations with similar results are shown). (***p≤0.001, **p<0.01, *p<0.05, ANOVA, Dunnett t-test, compared to MKN7+J99 without mucins.)

MKN7 cell viability after *H. pylori* infection was to different degrees protected by all mucin samples when added (at 50 µg/mL) to pre-cultures of *H. pylori* (Figure 8A). To a lesser degree, some of the mucin samples inhibited *H. pylori* binding to MKN7 cells (Figure 8B), but inhibition of *H. pylori* cell binding did not correlate to the ability of the mucins to bind the bacteria (see Figure 3) (*r* = −0.137, p = 0.725). The viability of the host cells is thus affected by other pathogen factors, such as the herein demonstrated mucin-induced changes in pathogen gene expression in addition to that mucins bind up bacteria and prevent them from adhering to the epithelial cells.

### Mucin-pretreatment of *H. pylori* can modulate cytokine production of gastric epithelial cells upon infection

**Figure 9:**
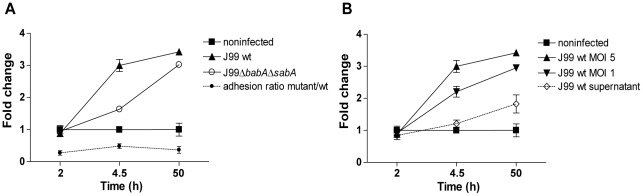
IL-8 production in response to *H. pylori* infection is concentration and adhesion dependent. A) Fold change of IL-8 concentration in cell culture media from MKN7 cells infected with *H. pylori* J99 wt and J99Δ*babA*Δ*sabA* (multiplicity of infection (MOI) of 5) compared to uninfected cells. The ratio of host cell binding of J99Δ*babA*Δ*sabA* to J99 wt is indicated in blue. B) Fold change of IL-8 concentration after infection with *H. pylori* J99 wt at a MOI of 5 and 1, and with *H. pylori* J99 wt supernatant, compared to uninfected cells.

Gastric epithelial MKN7 cells produced the pro-inflammatory cytokines IL-6 and IL-8 (Figure 7E), but not IL-1β, IL-10, IL-12p70 or TNF (data not shown), after infection with *H. pylori* J99. Pretreatment of J99 wt with the Le^b^ conjugate resulted in a decreased cytokine production (Figure 7E), reflecting less inflammatory response of the cells when fewer bacteria bind to the cells. Supporting the role of bacterial binding for the cytokine response, J99Δ*babA*Δ*sabA*, which bound less to MKN7 cells than J99 wt, induced less IL-8 production from the host cells than J99 wt (Figure 9A). Also, IL-8 production was dependent on the amount of bacteria added to the cells and live bacteria was more potent in inducing IL-8 compared to J99 wt culture supernatant (Figure 9B). Thus, when the bacterial factors are kept constant, IL-8 secretion increase with increasing *H. pylori* adhesion to the epithelial cells. However, when *H. pylori* were pretreated with mucin samples, cytokine production did not correlate to *H. pylori* host cell or mucin binding (*r* = −0.29-(−0.15), p = 0.38–0.71). Furthermore, pretreatment of *H. pylori* with the patient 1 soluble tumor mucins resulted in a gradual increase in production of both IL-6 and IL-8 with increasing mucin concentration by infected MKN7 cells (Figure 7E). If the level of adhesion to the host cells was the only determining factor for cytokine production one would have expected a decrease in cytokine production instead, as the mucin pre-treatment decreased the level of adhesion in an incrementing manner (Figure 7B). A higher IL-6 and IL-8 production was also detected after infection with *H. pylori* pretreated with patient 4 soluble tumor mucins already at 10 µg/mL (data not shown) and with three antral mucin samples at 50 µg/mL (Figure 8C). In addition, low levels of IL-1β and IL-12 was detected after *H. pylori* pretreatment with one of the mucin samples (patient 1 insoluble tumor mucins, high density, at 125 µg/mL). Other mucins tested did not interfere with the cytokine production (Figure 8C) and MKN7 cells cultured with only mucins were unaffected. Thus, although adhesion to host cells are of importance to induce host cell inflammatory signaling, mucins modulate this host cell signaling by additional means than by affecting *H. pylori* adhesion to its host.

## Discussion

Many studies have focused on the host response to *H. pylori*, but fewer studies have investigated how *H. pylori* is affected by the host and in particular by the host mucins. Previous studies report differences in mucin expression and glycosylation between individuals, tissue location and disease status [11], [13], [16]. Here we investigated how interindividual differences and infection associated changes in mucins, the building blocks of the mucus niche that *H. pylori* resides in and also the host's first line of defense, affect *H. pylori*. We show that these differences can change the interactions with *H. pylori*, causing modulations in proliferation and gene expression as well as alterations in how the bacteria affects the viability of host cells and host cell signaling. The regulation of *H. pylori* by mucins may be an important explanation for individual variations in host response and symptoms following infection.

We show that mucin binding ability via BabA seems to be an important factor for enhancing the *H. pylori* proliferation in response to mucins, whereas inhibition of proliferation in response to the mucins that have an inhibiting effect appears to be independent of adhesion to mucins. A previous study on bacterial growth in response to mucins showed that MUC2 inhibits proliferation of *C. jejuni* [21], which the authors suggest may be caused by adhesion of mucin oligosaccharide ligands to the bacterium, creating a physical barrier that limits its capability to obtain nutrients. Other studies have shown that porcine gastric mucin increases the growth of both *H. pylori* and *E. coli* [19], [20], [29], and Jiang et al. hypothesized that the increased *H. pylori* growth is due to mucin binding and thereby protection against the surrounding environment when bacteria are gathered around mucins [20]. We also think that aggregation of *H. pylori* around mucins would protect from the surrounding environment, both from the oxygen dissolved in culture media and from harmful acid, enzymes and immune factors in the gastric mucus layer. However, signaling between bacteria or between mucins and bacteria could also result in various responses affecting proliferation. Indeed, high cell density of *H. pylori* has been shown to benefit their proliferation [30], indicating bacteria-bacteria signaling when gathered around mucins to play a role for proliferation rates. The variation in bacterial cell density due to different growth rates of *H. pylori* strains may influence the degree of the effect mucins can have on proliferation after a certain culture time, as the ratio of bacteria to mucin will differ and bacteria may be differently susceptible at different growth rates.

Mucins are heavily glycosylated with a vast number of different glycans with unknown functions. The differences between how strains are affected by the same mucin sample are also explained by that the binding affinity of the adhesins can vary in their ability to recognize ligands depending on glycocontext and structural presentation [6], [31]. Moreover, adhesin repertoire differs between strains and only strains that bind via BabA and not the strain binding via SabA responded with a higher proliferation to the patient 1 soluble tumor mucins, which contain both Le^b^ and sialyl-Le^x^. The effect of the *H. pylori* ligands Le^b^ and sialyl-Le^x^ could be separately studied by co-cultures with glycoconjugates and recombinant mucin-type glycoproteins. The BabA ligand Le^b^ but not the SabA ligand sialyl-Le^x^ was able to induce a small, albeit statistically significant increase in *H. pylori* proliferation. The lack of large effects on *H. pylori* proliferation from glycoconjugates and recombinant glycoproteins could depend on glycocontext and presentation of the ligands, and that the carbohydrate content is much lower; 20–30% of the total molecular weight for HSA-glycoconjugates and around 40% for recombinant glycoproteins [32], compared to around 80% for mucins [33], resulting in less effect of multivalency and in total less glycans that can interact with *H. pylori*. However, even when we (over)compensate for differences in total glycan amounts by comparing recombinant protein/HSA-glycoconjugate at 50 µg/mL to human mucins at 10 µg/µL we still found approximately 10-fold larger effects of the human mucins.

The mechanism for effects of proliferation might not only be due to mucin binding as there are variations of the degree of stimulation not fully explained by binding ability. Kawakubo et al. show that α1,4-GlcNAc have antimicrobial affect on *H. pylori* [12]. Our results show that gland-derived mucins contain various amounts of this glycostructure and that some of the gland-derived mucins inhibit proliferation. However, there were also mucin samples that did not inhibit the proliferation although they contained α1,4-GlcNAc and most strains tested were either not or just slightly inhibited by gland mucins containing α1,4-GlcNAc. As we conclude that mucins enhance *H. pylori* proliferation and gene expression in an adhesion dependent manner, the total effect on *H. pylori* proliferation is likely to be the combined effects of mucins on gene expression, enhancing proliferation ability and antimicrobial effects of the α1,4-GlcNAc structure and there may be yet further mechanisms to be revealed.

There are two alternatives for how mucins can alter the growth curve of *H. pylori*: Either mucins can affect the length of the lag phase or the generation time at log phase. With most mucins in our experiment, *H. pylori* lag phase lasts 15 h similar as without mucins (Figure 1). The most exponential growth occurs between 20 and 40 h from start of experiment and at this time frame the growth curve slope of *H. pylori* with stimulatory mucins is steeper than that without mucins (Figure 1). Thus it seems that it is primarily the generation time that is altered by the interaction with mucins.

*H. pylori* were able to respond to the presence of mucins with an up-regulation (but never a down-regulation) of genes important in colonization and virulence processes. Changes in expression levels of different genes in response to the mucin samples correlated, indicating that some of the genes are regulated by the same pathways. All of the genes mentioned play a role in colonization and pathogenesis processes and a simultaneous up-regulation of these genes, as seen after culture with some of the mucin samples, may result in higher virulence of the bacteria. Expression levels of *cagA* correlated with decreased viability of host cells showing that some mucins can indeed induce virulence of *H. pylori*.

Expression of *cagA* correlated negatively to both proliferation and mucin binding. Similarly, *babA* expression was increased only in samples that do not stimulate proliferation and that, except from one sample, showed weak binding to *H. pylori*. Expression of *babA* and *cagA* correlated well in our experiments using one strain and variable external stimulation, and these genes have previously been shown to correlate in clinical isolates between different *H. pylori* strains [34]. Perhaps, when there is a low-degree of binding to mucins, *H. pylori* compensate by producing more BabA and CagA in order to keep an intermediate level of expression for successful long-term infection. Mucins may not up-regulate *cagA* expression in J99Δ*babA*Δ*sabA* either because binding interaction to the mucins is needed, even just to a low degree, to elicit the response or because BabA is involved in the regulation of *cagA* expression. A strong mucin binding also do not increase *babA* expression and may not be favorable as bacteria then can be washed away along with shedding mucins. Indeed, Rhesus monkeys with mucins that bind *H. pylori* better have lower infection density than monkeys having mucins with less BabA-ligands [11]. It has also been reported that low producers of BabA are associated with more severe clinical outcomes compared to high producers and BabA-negative strains [2]. At first glance, these studies may seem contrasting to our results that more mucin binding results in more *H. pylori* proliferation, which in theory may lead to higher infection density and more severe symptoms. However, mucins appear able to bind up also the resulting higher number of *H. pylori*, as is indicated by our observations of aggregation of bacteria around the proliferation-stimulating mucins and the increased inhibition of host cell binding simultaneously as increased *H. pylori* proliferation with higher concentration of proliferation-stimulating mucins, which protected host cell viability (see Figure 7A–C).

Previously published results suggest that BabA-mediated binding to the host cells facilitates translocation of CagA, which triggers a cytokine response [4]. Likewise, our data show that when the bacterial factors are kept constant, adhesion to host cells increases the cytokine response. As CagA has been shown to be important for IL-8 secretion, decreased production of CagA associated with increased proliferation in response to mucins further adds to the discrepancy with increased, instead of decreased cytokine secretion after mucin pre-treatment. Although the viability of host cells is protected by mucins that inhibit *H. pylori* binding to host cell, some mucins increased the cytokine response to *H. pylori*. This indicates that the host cells also can respond to unbound bacteria, but then without a major decrease in viability, and that the response is mediated through *H. pylori* factors affected by mucins other than CagA.

There seems to be a balance act between mucins trying to protect the host cells and *H. pylori* trying to adapt to the environment and mucins from most individuals induce some effects on *H. pylori* that appear protective for the host and some effects that appear harmful. Few mucin samples clearly tip the effect on *H. pylori* towards either protective or virulence-inducing. The patient 1 soluble tumor mucin seems to be the mucin sample inducing most virulence; increasing *H. pylori* proliferation, *cagA* expression and host cell cytokine production and showing relatively low ability of inhibiting host cell binding and protecting viability. If these effects contributed to tumor development or is a consequence thereof is unclear. Mucins that inhibit *H. pylori* proliferation, i.e. the mucins from glands and healthy tissue in our study, seem to be most efficient in protecting the host, as this would lead to fewer colonizing bacteria and as the damaging affect on *H. pylori* results in less host cell binding and protection of host cell viability. The inhibiting effects of some gland mucins may be a contributing factor to the low levels of *H. pylori* colonization found in gland mucosa [12], however, as all gland derived mucins in the present study did not have this effect there are likely additional reasons for lack of *H. pylori* colonization of the mucosal glands. The mucins isolated from healthy tissue either may result in more resistance to *H. pylori* or they have just not been modified by infection, as *H. pylori* infection may induce changes on mucins that are more favorable for colonization than the mucins in the healthy uninfected stomach. However, our study contained too few samples from either disease state to draw any firm conclusions regarding differences of effects on *H. pylori* by mucins from cancer-affected versus healthy stomachs.

To control for the possibility that we were not able to remove all GuHCl used in the purification of the mucins by dialysis, we also analyzed the effects of dialyzed 8 M GuHCl on the parameters, and found that at high concentrations this control also had some inhibiting effects on *H. pylori* proliferation and protected host cell viability, but not on the other parameters. However, most samples contained high concentrations of mucins and thereby the dialyzed samples were diluted to the extent that we did not detect any effects of the 8 M GuHCl dialysis control. The samples that had a low concentration of mucins and thereby were diluted so little that they may contain enough traces of GuHCl to have an effect on *H. pylori* was sample P10 and P6 TA-AS when used at 50 µg/mL. However, although sample P6 TA-AS should contain more GuHCl traces than the other samples it still stimulated proliferation of *H. pylori* J99 and the effect of mucins on *H. pylori* proliferation thus can exceed an effect of GuHCl.

In conclusion, mucins derived from individuals with or without gastric cancer affect *H. pylori* proliferation and gene expression, as well as interactions with host cells. Our results demonstrate a dynamic interplay between the bacterium and its host. Disease-dependent differences may occur, but our main conclusion is that there are differences between how mucins from different individuals affect *H. pylori*. Host-specific effects on *H. pylori* may explain the large individual differences in sensitivity to this infection and mucins thus have the potential of being active determinants of disease outcome.

## Materials and Methods

### Ethics statement

Gastric specimens were obtained after informed consent and approval of local ethics committees (IMIM-Hospital del Mar, Barcelona, Spain and Lund University Hospital, Lund, Sweden). For recently collected specimens, we have written informed consent statements, but some specimens were collected in 1996, i.e. over 15 years ago, at other institutions than where we are active and some individuals responsible for the collection are now retired. We were therefore not able to locate the patient consent forms for all patients in the study. We know that the specimens were collected after informed consent and after approval of the ethics committees as the individuals who were responsible for collecting these samples have written that in the statements of previously published articles using these samples (i.e. [6]).

### Isolation of mucins

**Figure 10:**
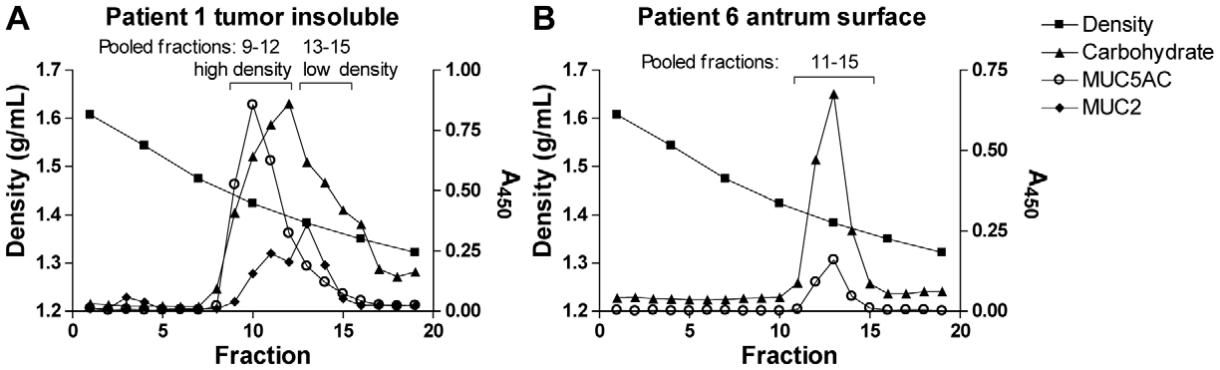
Isolation of mucins from gastric tissue. Panels show carbohydrate, MUC5AC and MUC2 content in density gradient fractions diluted 1∶500. The fractions for all samples were pooled based on this type of graphs, and in these graphs the pooled fractions are indicated by brackets. A) Density gradient of the patient 1 insoluble tumor mucin sample, which is pooled into two samples with most MUC5AC in the high density sample and most MUC2 in the low density sample. B) Density gradient of the patient 6 tumor-adjacent antrum surface sample.

Mucins were isolated from archived gastric specimens. Four of the specimens were from gastric adenocarcinoma tumors (intestinal type) and another four were from macroscopically normal mucosa of tumor-affected stomachs, as evaluated by a clinical pathologist. These specimens of normal gastric mucosa isolated from tissues adjacent to tumors were sufficiently large to allow this material to be divided, prior to mucin isolation, into surface versus gland material. Additionally, three specimens were from full gastric wall of patients who underwent elective surgery for morbid obesity. They had no history of peptic ulcer disease, and their stomachs were macroscopically normal at the time of operation. Specimens (approximately 1.5×1.5 cm) were taken from fundus and antrum of the stomach [6]. The samples are summarized in Table 1. The mucins were isolated from tissue specimens after rinsing, which removes the extracellular mucus layer, and the mucins isolated thus comprise the intracellular mucins that have not been degraded by extracellular processes. Mucins were isolated by isopycnic density gradient centrifugation from these materials as previously described [35]. Mucins soluble in guanidinium hydrochloride (GuHCl) were separated from insoluble mucins, which were later solubilized by reduction and alkylation of disulphide bonds. As the insoluble samples from non-tumor tissue did not contain MUC2 or other mucins, these were excluded from the study. Density gradient fractions of purified mucin samples were analyzed for carbohydrates as periodate-oxidisable structures in a microtiter-based assay as previously described (Figure 10) [28] and sialic acid was detected using an automated periodate-resorcinol method [36]. Density measurements were performed using a Carlsberg pipette as a pycnometer and DNA was detected by UV light absorbance at 280 nm.

### Preparation of mucin samples

Gradient fractions containing mucins were pooled together to obtain one sample for each specimen (Table 1, Figure 10). Gradients containing separated groups of fractions with different mucin and carbohydrate content were pooled into two samples (example in Figure 10A). All samples were dialyzed in phosphate buffered saline (PBS) to remove guanidinium hydrochloride and cesium chloride (CsCl) from the medium. Mucin concentration in pooled samples was determined by detection of carbohydrate as periodate-oxidisable structures in a microtiter-based assay: Flexible 96-well plates (BD Biosciences, Franklin Lakes, NJ, USA) were coated with mucin sample and left overnight at 4°C. After washing three times with washing solution (5 mM Tris-HCl, 0.15 M NaCl, 0.005% Tween 20, 0.02% NaN_3_, pH 7.75), the carbohydrates were oxidized by treatment with 25 mM sodium metaperiodate in 0.1 M sodium acetate buffer, pH 5.5 for 20 min in room temperature. The plates were washed again and the wells were blocked with DELFIA blocking solution (50 mM Tris-HCl, 0.15 M NaCl, 90 µM CaCl_2_, 4 µM EDTA, 0.02% NaN_3_, 6% sorbitol, 0.1% BSA, pH 7.75) for 1 h. After further washing steps, the samples were incubated for 1 h with 2.5 µM biotin hydrazide in 0.1 M sodium acetate buffer, pH 5.5, followed by washing again. Europium-labeled streptavidin was diluted 1∶400 in assay buffer (50 mM Tris-HCl, 0.15 M NaCl, 20 µM DTPA, 0.01% Tween 20, 0.02% NaN_3_, 1.5% BSA, pH 7.75) and was added to the wells. After 1 h incubation, the plates were washed six times and then incubated with enhancement solution (0.05 M NaOH, 0.1 M ftalat, 0.1% Triton X-100, 50 µM TOPO, 15 µM β-NTA) for 5 min on a shaker. The plates were measured using Wallac 1420 VICTOR^2^ plate reader with the Europium label protocol (PerkinElmer, Waltham, MA, USA). The concentrations were calculated from a standard curve of a fusion protein of MUC1, 16TR and IgG2a Fc starting at a concentration of 20 µg/mL and using seven 1∶2 serial dilutions [37]. This method of concentration determination was chosen as all mucins do not come into solution after freeze drying, and determining concentration by freeze drying therefore can contain large errors as well as remove mucin species selectively.

### Enzyme-linked immunosorbent assays

Mucins and carbohydrates in separate gradient fractions were detected by antibodies in enzyme-linked immunosorbent assays (ELISA) as described previously [28]. Pooled mucins gradient samples were analyzed for the relative content of Le^b^, sialyl-Le^a^, sialyl-Le^x^, α1,4-GlcNAc, MUC5AC, MUC6, MUC2 and MUC5B. Mucin samples were diluted in 4 M GuHCl to 6 µg/mL for the glycosylation analysis or to 3 µg/mL for the mucin analysis and coated on to 96-well polysorb plates (NUNC A/S, Roskilde, Denmark) over night at 4°C. The samples to be analyzed with LUM6-3, LUM2-3 and LUM5B-2 were reduced with 2 mM 1,4-dithiothreitol in 6 M GuHCl, 5 mM sodium EDTA, 0.1 M Tris-HCl buffer, pH 8.0, at 37°C for 1 h and alkylated in 5 mM iodacetamide at room temperature for 1 h in the dark to expose the epitopes. All plates were washed 3 times with PBS 0.05% Tween (washing buffer) and the wells were blocked for 1 hour with Blocking Reagent for ELISA containing 0.05% Tween (blocking buffer) in room temperature. After discarding the blocking buffer, the plates were incubated for 1 hour with primary antibody for glycan structure; Seraclone anti-Le^b^ (clone LE2, Biotest, Dreieich, Germany), anti-sialyl-Le^a^ (clone CA19-9, NeoMarkers, Freemony, CA, USA), anti-sialyl-Le^x^ (AM3, gift from Dr C. Hanski, University Medical Center Charité, Berlin, Germany) diluted to 1 mg/mL, 1∶200, 1∶1000 and 1∶20, respectively, and anti-α1,4-GlcNAc (HIK1083, Kanto Chemical Co., Inc.,Tokyo, Japan) diluted 1∶50, and for mucin; anti-MUC5AC (45M1, Sigma-Aldrich, St. Louise, MO, USA) diluted 1∶8000 and anti-MUC6 (LUM6-3) [35], anti-MUC2 (LUM2-3) [38] and anti-MUC5B (LUM5B-2) [39] diluted 1∶2000 in blocking buffer. The plates were again washed 3 times and then incubated for 1 hour with 0.8 µg/mL horse radish peroxidase (HRP) conjugated anti-mouse IgM, anti-mouse IgG or anti-rabbit IgG (Jackson ImmunoResearch Laboratories, Inc., West Grove, PA, USA) diluted in blocking buffer. After further washing, tetramethylbenzidine (TMB) substrate (EMD Biosciences, Inc., San Diego, CA, USA) was added and the plates were monitored for color development. The reaction was stopped with an equivalent amount of 0.5 M H_2_SO_4_ and the absorbance at 450 nm was measured.

### Bacterial culture conditions and strains

*H. pylori* strains were cultured on *Brucella* agar (Oxoid, Basingstoke, Hampshire, England) supplemented with 10% bovine blood, 1% IsoVitox (Oxoid), 4 mg/L Amphotericin B, 10 mg/L Vancomycin and 5 mg/L Trimethoprim in 5% O_2_ and 15% CO_2_ at 37°C. Eight *H. pylori* strains/isogenic mutant with known binding specificity were used in this study: *H. pylori* strains J99 wild type (wt) bind Le^b^ and sialyl-Le^x^, whereas the isogenic J99 adhesion mutant lacking the BabA and SabA adhesins (J99*babA*::cam*sabA*::kan, referred to as J99Δ*babA*Δ*sabA*), do not bind Le^b^ or sialyl-Le^x^ [10] (kindly provided by Prof. Thomas Borén, Umeå University, Sweden). *H. pylori* 26695 also do not bind Le^b^ or sialyl-Le^x^, whereas CCUG17875/Le^b^ bind Le^b^ but not sialyl-Le^x^ and CCUG17874 bind sialyl-Le^x^ but not Le^b^ [6] (kindly provided by Prof. Susann Teneberg, University of Gothenburg, Sweden). The clinically isolated strains HP201 and HP1172, but not HP364, bind mucins positive for Le^b^ and sialyl-Le^x^ [28] (kindly provided by Prof. Lars Engstrand, Swedish Institute for Infectious Disease Control, Sweden).

### *H. pylori* binding to mucins

Biotinylation of bacteria was performed as previously described [6]. Binding of biotinylated *H. pylori* J99 wt to mucins samples were assessed using a microtiter-based assay. Mucin samples were diluted in 4 M GuHCl to 6 µg/mL and coated on 96-well polysorb plates over night at 4°C. Subsequent steps were performed as described previously [28]. The plates were washed 3 times with washing buffer and the wells were blocked for 1 hour with blocking buffer. After discarding the blocking buffer, biotinylated bacteria with an OD_600_ of 0.1 were diluted 1∶20 in blocking buffer, pH 7.5, and added to the plates, which then were incubated in a bacterial shaker at 37°C for 2 hours. The plates were washed 3 times and then incubated for 1 hour in room temperature with blocking buffer containing 1 µg/mL of horse radish peroxidase conjugated streptavidin. After further washing steps, TMB substrate was added and the plates were incubated for 25 min. The reaction was stopped with an equivalent amount of 0.5 M H_2_SO_4_ and the plates were read in a microplate reader at 450 nm after color stabilization.

### Proliferation assay

*H. pylori* were harvested from *Brucella* plates into PBS. Harvested bacteria at an OD_600_ of 0.3 were cultured in brain heart infusion medium (BHI) with 10% fetal bovine serum (FBS) with purified and dialyzed mucin samples diluted in PBS at 10, 50 and 125 µg/mL of mucin. *H. pylori* were also culture with 50 µg/mL of glycoconjugates of the carbohydrate structures Le^b^ and sialyl-Le^x^ coupled to human serum albumin (HSA) to create multivalency (IsoSep AB, Tullinge, Sweden) or with 50 µg/mL of the recombinant mucin-type glycoprotein P-selectin glycoprotein ligand-1 (PSGL-1) produced in CHO-K1 cells; unmodified CHO-K1 mucin containing mono- and disialylated core 1 (CM), sialyl-Le^x^ substituted mucin (CM-SLex), Le^b^ substituted mucin (CM-Leb) and blood group A active mucin (CM-A) [26], [27], and with a mannose-containing mucin lacking sialic acid produced in the yeast Pichia pastoris (PPM) [25]. A control for normal proliferation was obtained by adding only PBS without mucins, or HSA (Sigma) without conjugates, to the culture medium, and a control for efficient removal of toxic material in dialysis was obtained by adding dialyzed guanidinium hydrochloride diluted in PBS to the culture medium. Bacteria were cultured in a total volume of 100 µL in 96-well plates, 12 to 18 replicates, for 24 or 60 hours at 37°C under aerobic conditions (10% CO_2_). The strains used in this study grow well both at microaerobic and aerobic conditions. Optical density (OD) at 560 nm was measured at time points throughout the culturing. At the end of the incubation, bacteria from a subset of wells were cultured on *Brucella* plates and the number of colony forming units (CFU) was calculated after 5 days of incubation. The CFU counts obtained were proportional to the optical density at 560 nm, and therefore, OD at 560 nm is presented in the graphs.

### Fluorescence microscopy

For visualization, *H. pylori* were stained with a LIVE/DEAD® BacLight™ bacterial viability kit (Molecular Probes, Leiden, The Netherlands) according to the manufacturer's directions. Bacteria from two replicate wells were pooled after 37 h proliferation with mucin samples or glycoconjugates and washed twice with 0.85% NaCl and centrifuged at 2500 g for 3 min between the washes. Washed pellets were resuspended with 100 µL 0.85% NaCl and incubated with 0.3 µL premixed staining components for 15 min in the dark. Stained bacteria were applied to a microscopy slide and studied immediately under a fluorescence microscope for red and green fluorescence simultaneously.

### RNA extraction and cDNA synthesis

After 24 h of proliferation in the presence of mucins, bacterial replicates were pooled into microcentrifuge tubes, centrifuged at 3000 g for 4 min and resuspended in RNA*later*® (Applied Biosystems, Foster City, CA, USA). The samples were incubated over night at 4°C and then stored at −20°C. Bacteria stored in RNA*later*® were centrifuged at 5000 g for 5 min. The pellet was resuspended with 100 µL of 400 µg/mL lysozyme in TE buffer (1 mM EDTA, 10 mM Tris, pH8) and incubated at room temperature for 5 min. RNA extraction was continued with Qiagen's RNeasy kit (Qiagen GmbH, Hilden, Germany), including DNase treatment with Qiagen's RNase-Free DNase Set. Extracted RNA was eluted in 30 µL RNase-free water, and stored at −70°C. Concentration of RNA was determined by measuring the optical density at 260 nm with a spectrophotometer (NanoDrop Technologies, Wilmington, DE, USA). cDNA was synthesized from 400 ng RNA using Quantitect Reverse Transcription kit (Qiagen). Each sample was DNase treated (included in the kit) and divided into two aliquots, one was used to transcribe cDNA while the other was used as the reverse transcription negative control in the subsequent real-time PCR assays. The reverse transcription PCR protocol followed two steps: reverse transcription for 15 min at 42°C and inactivation of enzyme for 3 min at 92°C. The reaction was carried out in a total volume of 20 µL: 7 µL sample containing 400 ng RNA together with 1 µL random hexamer primers, 1 µL reverse transcriptase (RT), 4 µL RT buffer and 7 µL water (nucleotide, DNase, and RNase free) or with just 13 µL water in the negative control. cDNA was stored at −20°C.

### Primer design

Gene sequences of *H. pylori* strain J99 (GenBank accession number AE001439) were retrieved from the NCBI nucleotide database (www.ncbi.nlm.nih.gov). Primer pairs were designed using Primer3 web-based program (http://frodo.wi.mit.edu/primer3/) with default settings. Gene specificity of primers was confirmed using NCBI nucleotide BLAST. Previously described primer sequences were used for *cagA*, *flaA*, *ureA* [40]. All primer sequences used are listed in table 5.

**Table 5:** Primers used in real-time PCR.

Gene	Full name	Direction	Sequence	Reference
*jhpr6*	16S ribosomal RNA	Forward	TCGGATTGTAGGCTGCAACTC	
		Reverse	CCGCAACATGGCTGATTTG	
*babA*	Blood group antigen binding adhesin A	Forward	GGAAGCGAAAGTTTGAGTGG	
		Reverse	GAGAGGCTTAGCGGGACTTT	
*cagA*	Cytotoxin-associated gene A	Forward	TGATGGCGTGATGTTTGTTGA	[40]
		Reverse	TCTTGGAGGCGTTGGTGTATT	[40]
*clpA*	ATP-dependent Clp protease A	Forward	TGGCTAAAGAATTGGCCTTG	
		Reverse	AGGACTTCCGATGAGCTTTG	
*flaA*	Flagellin A	Forward	ATGACGGTGGCGGATTCTT	[40]
		Reverse	GATAATCCCCATGCCGTCATT	[40]
*sabA*	Sialic acid binding adhesin A	Forward	GAGCGTTGCTTACGGTTGAG	
		Reverse	CCCAACAAAACGCTACCACT	
*ureA*	Urease subunit A	Forward	TATGGAAGAAGCGAGAGCTGGTA	[40]
		Reverse	GAGTGCGCCCTTCTTGCAT	[40]

### Real-time PCR

Real-time PCRs were run in 96-well plates (Applied Biosystems), in a total volume of 20 µL containing 10 µL SYBR green real-time PCR master mix (Applied Biosystems), 10 pmol of each primer, 6.5 µL water and 1.5 µL of a sample. Reverse transcription negative controls and non-template controls were included in each run. Amplification was performed using an ABI7500 real-time PCR instrument (Applied Biosystems) with default settings for the amplification protocol and including a dissociation step in the end of the program for melting temperature (*T*_m_) analysis to confirm amplification specificity. Expression was calculated with 2^−ΔCt^ and normalized against the 16S rRNA expression. Results are shown as fold-changes of mRNA expression in samples relative to mRNA expression levels in bacteria cultured without mucins. An expression difference of more than 4 fold from the PBS-treated control bacteria is regarded as an up- or down-regulation.

### MKN7 cell culture and infection with mucin treated *H. pylori*

The gastric epithelial cell line MKN7 is well-differentiated and form firmly adherent continuous cell layers expressing MUC1 on the apical side [24], [41], [42]. MKN7 cells were cultured in RPMI 1640 (Lonza, Verviers, Belgium) containing 10% FBS, 100 units/mL penicillin G sodium and 100 µg/mL streptomycin. The medium was changed to antibiotic-free medium 24 h prior to infection experiments. The cells were incubated at 37°C and 5% CO_2_. Prior to infection, MKN7 cells were seeded at a density of 4×10^4^ cells/well in 96-well plates and incubated for 3 days, at which time point they were confluent. *H. pylori* J99 wt was cultured for 24 h in 100 µL antibiotic-free cell culture medium in the presence of mucins prepared as above. The media on the confluent MKN7 cells was substituted with the media containing mucin-treated *H. pylori* at a density of 3×10^5^ bacteria/well (multiplicity of infection (MOI) of 5). The MKN7 *H. pylori* co-culture was incubated for 24 h. All incubations were performed at 37°C in aerobic conditions.

### Cell viability and apoptosis assays

The viability of MKN7 cells infected with *H. pylori* was evaluated using the WST-1 cell proliferation assay (TaKaRa Bio Inc.,Otsu, Shiga, Japan) measuring metabolic activity. Half of the media was transferred to other 96-well plates to be measured as the background from media and bacteria not attached to the cells. Both plates (with and without MKN7 cells) were incubated with 4.5 µL/well of WST-1 reagent for 1 h at 37°C. The plates were put on a shaker for 1 min and then measured at 450 nm in a microplate reader. The values for the MKN7 cells were reduced with the background values from the bacteria, which ranged from −0.004 to 0.02. Cell viability was also analyzed by flow cytometry using Annexin V-PE Apoptosis Detection Kit No. 1 (BD Pharmingen) according to the manufacturer's instructions. For this experiment, non-adherent cells in the media and attached cells harvested with trypsin from four replicates were pooled to one sample.

### Assessment of mucin-treated *H. pylori* binding to MKN7 cells

Infected MKN7 cells used for the WST-1 assay were subsequently washed three times with PBS, fixed with 4% formaldehyde for 10 min on ice, and then incubated with 70% ethanol at 4°C over night. The plates were washed 3 times with washing buffer and the wells were blocked for 1 hour with blocking buffer in room temperature. To detect bound bacteria, the plates were then incubated for 1 hour with rabbit anti-*H. pylori* serum (kindly provided by Prof. Thomas Borén, Umeå university, Sweden) diluted 1∶1500 in blocking buffer. The plates were again washed 3 times and then incubated for 1 hour with HRP conjugated anti-rabbit IgG (Jackson ImmunoResearch Laboratories, Inc.) diluted to 0.8 µg/mL in blocking buffer. After further washing, TMB substrate was added and the reactions were stopped with an equivalent amount of 0.5 M H_2_SO_4_ after equal time of incubation. The plates were read in a microplate reader at 450 nm.

### Cytokine analysis

Culture supernatant from 6 replicates of infected MKN7 cells were pooled to one sample and diluted 1∶10. Cytokine production was quantified using the CBA Human Inflammatory Cytokines Kit (BD Biosciences) according to the manufacturer's instructions. The samples were analyzed on a FACSCalibur™ (BD Biosciences). In addition, IL-8 was analysed in supernatants from cell cultures using the BD OptEIA Human IL-8 ELISA Set (BD Biosciences) according to manufacturer's instructions.

### Statistics

Statistical analyses were performed using SPSS Statistics 17.0 (SPSS Inc, Chicago, IL, USA) software package. The level of significance was set at p<0.05.
